# Association between metabolic syndrome and risk of incident low back pain: A retrospective cohort study using real-world data from Japan

**DOI:** 10.1016/j.pmedr.2025.103162

**Published:** 2025-07-04

**Authors:** Shinsuke Okawa, Takuya Yamada, Mari Irie, Kumi Sugimoto, Yoshiharu Fukuda

**Affiliations:** Teikyo University Graduate School of Public Health, Itabashi-Ku, Tokyo, Japan

**Keywords:** Low back pain, Metabolic syndrome, Real-world data, Health checkup, Health insurance claims

## Abstract

**Objectives:**

Obesity is a well-established risk factor for low back pain (LBP). Emerging evidence suggests that other metabolic abnormalities may also be associated with LBP; however, the association between metabolic syndrome (MetS) and LBP remains unclear. This study assessed the association of MetS with the risk of incident LBP using longitudinal real-world data from Japan.

**Methods:**

Health insurance claims and health checkup data from prefectural government employees across all Japanese prefectures except Tokyo (April 1, 2018–March 31, 2023) were linked. Incident LBP was identified from claims, and MetS status from checkups. Participants who underwent a health checkup between April 1, 2018 and March 31, 2019 with no LBP that year were followed from April 1, 2019 to March 31, 2023. Cox proportional hazards models estimated hazard ratios (HRs) and 95 % confidence intervals (CIs) to explore the association of MetS with LBP risk.

**Results:**

Among 111,095 participants (mean follow-up: 3.3 years, 362,911.7 person-years), 16,894 developed LBP (15.2 %). MetS was associated with a significantly higher LBP risk (HR = 1.24; 95 % CI, 1.19–1.30). In sex-stratified analyses, the HR was 1.23 (95 % CI, 1.17–1.29) for males and 1.34 (95 % CI, 1.17–1.54) for females. Participants with abdominal obesity with additional MetS components had a greater LBP risk than those with abdominal obesity alone.

**Conclusion:**

MetS may be a predictor of incident LBP, highlighting the potential value of MetS management in LBP prevention.

## Highlights

• Metabolic syndrome was associated with an increased risk of incident low back pain.
• Abdominal obesity with additional metabolic components increased low back pain risk.
• Analysis of real-world data enabled longitudinal estimation of low back pain risk.

## 1 Introduction

Low back pain (LBP) is one of the most prevalent musculoskeletal disorders worldwide, affecting an estimated 619 million people in 2020 (GBD 2021 Low Back Pain Collaborators, 2023). The condition has a global point prevalence of 11.9 % and a one-month prevalence of 23.2 %, with a notably higher prevalence in females (Hoy et al., 2012). LBP is also a major contributor to years lived with disability (GBD 2021 Low Back Pain Collaborators, 2023) and imposes a significant social and economic burden, including healthcare costs (Dagenais et al., 2008), premature retirement (Schofield et al., 2008), and reduced workplace productivity (Carregaro and Tottoli, 2020). Consequently, preventing LBP is a critical public health issue.

Previous studies have identified occupational ergonomic factors (Steffens et al., 2015; Wai et al., 2010), smoking (Shiri et al., 2010a; Shiri and Falah-Hassani, 2016), and obesity (Elgaeva et al., 2020; Lean et al., 1998; Shiri et al., 2010b) as risk factors for LBP. Regarding obesity, LBP has been linked to body mass index (BMI) (Elgaeva et al., 2020; Shiri et al., 2010b) and waist circumference (Lean et al., 1998), both commonly used indicators. Notably, abdominal obesity has shown a stronger association with LBP than systemic obesity (Shiri et al., 2008). The underlying mechanisms include increased mechanical load on the musculoskeletal system (Rodriguez-Martinez et al., 2016; Singh et al., 2015), physical inactivity (Shiri et al., 2013; Smuck et al., 2014), and inflammation due to excessive fat accumulation (Briggs et al., 2013; Shoelson et al., 2007).

Metabolic syndrome (MetS) is characterized by a combination of abdominal obesity and metabolic abnormalities, including hypertension, dyslipidemia, and impaired glucose tolerance, which increase the risk of cardiovascular disease (Eckel et al., 2005; Rochlani et al., 2017). Recently, MetS has also been linked to an elevated risk of musculoskeletal disorders (Li et al., 2016; Mäntyselkä et al., 2010; Pan et al., 2020). However, evidence regarding its effect on LBP remains limited. Most existing evidence is cross-sectional, which restricts causal inference. Three cross-sectional studies from Japan and the United Kingdom (Ono et al., 2012; Perera et al., 2022; Yoshimoto et al., 2019) found an association between MetS and LBP only in females. By contrast, a study from China (Huang et al., 2024) reported a negative association in its cross-sectional analysis, whereas its longitudinal analysis found no significant association. To date, only one longitudinal investigation (Huang et al., 2024) is available, and its generalizability may be limited given that its participants with MetS reported healthier behaviors than those without MetS. In addition, most previous studies defined LBP using self-reported measures (Ha, 2011); however, to our knowledge, no study has yet examined the association between MetS and LBP using claims-based diagnoses. Finally, evidence on how LBP risk varies according to different MetS component combinations remains scarce (Perera et al., 2022; Yoshimoto et al., 2019).

In Japan, the use of real-world data (RWD), including insurance claims and health checkup data from public medical insurers, has facilitated new research advancements (Sugimoto et al., 2024). Multi-year data allow for longitudinal analysis (Imaizumi et al., 2022; Ma et al., 2023), generating more robust evidence. By leveraging RWD, researchers can track large populations over extended periods, improving the accuracy of assessments regarding the relationship between LBP and MetS.

This study aimed to assess the association of MetS with the risk of incident LBP. Specifically, we conducted a longitudinal analysis among middle-aged Japanese workers using large-scale insurance claims and health checkup data.

## 2 Methods

### 2.1 Study design and data sources

This retrospective cohort study analyzed insurance claims and health checkup data collected between April 1, 2018 and March 31, 2023, corresponding to Japanese fiscal years 2018–2022. The data were provided by health insurance associations for prefectural government employees across all prefectures of Japan except Tokyo as employees of the Tokyo Metropolitan Government are insured through a separate association and we did not have access to their data. Insurance claims data included information on diagnoses and treatment start dates, while health checkup data comprised all items from the Specific Health Checkup (SHC), an annual screening program mandated for insurers in Japan to prevent MetS and lifestyle-related diseases (Ministry of Health, Labour and Welfare, 2025a). The SHC includes measurements such as waist circumference, blood pressure, lipid profile, and glucose levels. The baseline period was April 1, 2018–March 31, 2019 (fiscal year 2018) and the follow-up period was from April 1, 2019–March 31, 2023 (fiscal years 2019–2022).

### 2.2 Study population

Participants who underwent the SHC in fiscal year 2018 were identified from health checkup data. The following exclusion criteria were applied: loss of health insurance eligibility during fiscal year 2018, death during fiscal year 2018, failure to undergo the SHC, age ≥ 65 years, dependent insurance status (e.g., spouse or family member), missing or outlier MetS data, missing health behavior data, and LBP claims in fiscal year 2018. A total of 260,429 individuals were eligible for the SHC, of whom 149,334 were excluded; thus, 111,095 participants remained for analysis.

In Japan, all prefectural government employees, except those in Tokyo, are covered by the same employment-based health insurance scheme, which remains valid until retirement. The statutory retirement age for prefectural employees ranges from 60 and 65 years. To guarantee adequate follow-up, we therefore excluded individuals who were aged 65 years or older at baseline.

Outliers in laboratory data were defined as follows: waist circumference < 30 cm or ≥ 200 cm, systolic blood pressure (SBP) <50 mmHg or ≥ 250 mmHg, diastolic blood pressure (DBP) <30 mmHg or ≥ 150 mmHg, triglycerides <20 mg/dL or ≥ 2000 mg/dL, high-density lipoprotein cholesterol (HDL—C) <10 mg/dL or ≥ 200 mg/dL, and fasting blood glucose (FBG) <30 mg/dL. These thresholds, based on prior studies, excluded participants with implausible values due to input errors or similar issues (Fukuda, 2011; Sugimoto et al., 2024).

### 2.3 Exposure: Metabolic syndrome

MetS was defined according to the criteria established by the Metabolic Syndrome Diagnostic Criteria Committee in Japan (Metabolic Syndrome Diagnostic Criteria Review Committee, 2005). It was characterized by abdominal obesity (waist circumference ≥ 85 cm in males or ≥ 90 cm in females) and at least two of the following three metabolic factors: hypertension (SBP ≥130 mmHg and/or DBP ≥85 mmHg and/or medication use for hypertension), hyperlipidemia (triglycerides ≥150 mg/dL and/or HDL-C < 40 mg/dL and/or medication use for dyslipidemia), and hyperglycemia (FBG ≥110 mg/dL and/or medication use for diabetes).

Participants were classified into five mutually exclusive and collectively exhaustive groups according to abdominal obesity and the number of additional MetS components, a system adopted from a previous study (Yoshimoto et al., 2019): (1) no MetS components; (2) MetS components excluding abdominal obesity; (3) abdominal obesity only; (4) abdominal obesity with one additional MetS component; and (5) abdominal obesity with two or more MetS components. Definitions of these categories are provided in Supplementary Table 1. No participants were excluded; all were retained for the final analysis.

### 2.4 Outcome: Low back pain

The outcome was defined as a diagnosis of LBP in the insurance claims data. Participants with LBP were identified using the following ICD-10 codes: M54.3 (sciatica), M54.4 (low back pain with sciatica), and M54.5 (low back pain) (GBD 2021 Low Back Pain Collaborators, 2023). The study endpoint was incident LBP during the follow-up period.

### 2.5 Covariates

Age, sex, and health behaviors were considered potential confounders. Health behaviors, assessed using standard SHC questions (Ministry of Health, Labour and Welfare, 2018), included smoking, insufficient physical activity, and alcohol consumption. Definitions were as follows: Current smoking, having smoked at least 100 cigarettes in total or for at least six months, and having smoked within the past month; Insufficient physical activity, not engaging in at least 30 min per session of exercise inducing light sweating at least twice per week for at least one year and/or not walking or performing an equivalent activity in daily life for at least one hour per day; Alcohol consumption, drinking alcohol every day. The SHC questionnaire does not distinguish between those who never smoked and former smokers. For alcohol, the response options are “daily,” “occasionally,” and “rarely/never;” to avoid ambiguity, we used only the “daily” category in the present analysis.

### 2.6 Statistical analysis

Participants at baseline were stratified by MetS status, and their characteristics were summarized. Because of the very large sample size, normality was assessed for all continuous variables using histograms and quantile–quantile plots. Categorical variables are presented as frequencies and percentages. Continuous variables are reported as means ± standard deviation for normally distributed data and as medians with interquartile ranges for non-normally distributed data. Group differences for normally distributed continuous variables were analyzed with independent *t*-tests or one-way analysis of variance; when normality was not satisfied, the Mann-Whitney *U* test or Kruskal-Wallis test was used. Categorical variables were compared with the chi-square test.

A Cox proportional hazards model was employed for survival analysis to estimate hazard ratios (HRs) and 95 % confidence intervals (CIs) for the association between MetS and LBP risk. An interaction term (MetS × sex) was added to test whether the association differed between males and females. The same model was also applied to each individual MetS component—hypertension, hyperlipidemia, and hyperglycemia—to obtain component-specific HRs for LBP. The diagnosis of LBP was defined as the event, with survival time measured from the end of the baseline period (April 1, 2019) until event occurrence or censoring due to loss of health insurance eligibility or death. The event date corresponded to the first recorded LBP treatment in the claims data, and the censoring date was the day before the loss of health insurance eligibility, or, for deaths, it was the first day of the fiscal year (April 1) in which the death occurred, because the exact date of death is not available in the database. Cumulative incidence during the follow-up period was illustrated as Kaplan–Meier curves, and differences between participants with and without MetS were tested with log-rank tests. Sex, age, and health behaviors were adjusted for; therefore, all subsequent HRs refer to estimates from the fully adjusted model. The proportional hazards assumption was tested using Schoenfeld residuals in every model, and it was not violated overall (global *p* > 0.05). Sensitivity analysis included (1) a six-month lag after baseline, (2) redefining LBP by excluding sciatica (ICD-10 M54.3), (3) applying sex-specific HDL-C cut-offs (< 40 mg/dL for men; < 50 mg/dL for women) corresponding to international diagnostic criteria (Alberti et al., 2009), and (4) evaluating the age × MetS interaction. All statistical analyses were conducted using R software (version 4.4.2). Statistical significance was set at a two-sided α = 0.05 (5 %) for main effects and α = 0.10 (10 %) for interaction terms, reflecting the lower statistical power of interaction tests.

### 2.7 Ethical considerations

This study adhered to the Declaration of Helsinki and was approved by the Teikyo University Ethics Review Committee (Teirin 18–200-6, approval date: January 20, 2025).

## 3 Results

**Fig. 1:**
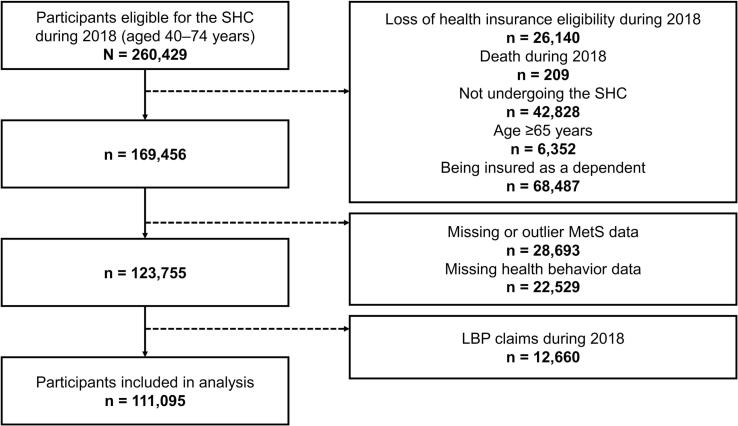
Study flowchart of participants eligible for the Specific Health Checkup in fiscal year 2018, based on nationwide insurance claims and health checkup data for prefectural government employees in Japan. Abbreviations: SHC, specific health checkup; MetS, metabolic syndrome; LBP, low back pain.

Between April 1, 2018 and March 31, 2019, 260,429 individuals were eligible for the SHC. Of these, 149,334 were excluded for the following reasons: loss of health insurance eligibility during fiscal year 2018 (26,140), death during fiscal year 2018 (209), not undergoing the SHC (42,828), being aged ≥65 years (6352), being insured as a dependent (68,487), or having missing or outlier MetS data or health behavior data (45,701). The number of individuals with missing MetS data, examination outliers, and missing health behavior data was 28,643 (11.0 %), 63 (0.02 %), and 22,529 (8.7 %), respectively. Additionally, 12,660 individuals with LBP claims during fiscal year 2018 were excluded, including 270 whose follow-up claims indicated that their initial diagnosis dated to fiscal year 2017 or earlier. Consequently, 111,095 participants without LBP were included in the analysis (Fig. 1).

Table 1 presents the characteristics of participants with and without MetS. The median age was 50.0 years (interquartile range 46.0–55.0), and 29.3 % were female. Among participants, 15.0 % were current smokers, 87.5 % had insufficient physical activity, and 24.7 % consumed alcohol daily. The prevalence of MetS was 12.5 %. Regarding MetS components, 33.6 % had abdominal obesity, 35.1 % had hypertension, 25.4 % had hyperlipidemia, and 12.2 % had hyperglycemia. Supplementary Table 2 provides participant characteristics based on different MetS component combinations.

**Table 1:** Baseline characteristics of participants with and without metabolic syndrome among prefectural government employees in Japan who underwent the Specific Health Checkup in fiscal year 2018.

Variable	Overall (*n* = 111,095)	No MetS (*n* = 97,209)	MetS (*n* = 13,886)	*p*-value
Demographics				
Age – years a	50.0 (46.0–55.0)	50.0 (45.0–54.0)	53.0 (49.0–57.0)	<0.01^b^
Sex				<0.01^c^
Male	78,500 (70.7)	65,711 (67.6)	12,789 (92.1)	
Female	32,595 (29.3)	31,498 (32.4)	1097 (7.9)	
Metabolic Syndrome Components				
Abdominal obesity	37,369 (33.6)	23,483 (24.2)	13,886 (100.0)	<0.01^c^
Hypertension	38,992 (35.1)	26,381 (27.1)	12,611 (90.8)	<0.01^c^
Dyslipidemia	28,265 (25.4)	16,721 (17.2)	11,544 (83.1)	<0.01^c^
Hyperglycemia	13,511 (12.2)	6161 (6.3)	7350 (52.9)	<0.01^c^
Health Behaviors				
Smoking	16,693 (15.0)	13,515 (13.9)	3178 (22.9)	<0.01^c^
Physical inactivity	97,188 (87.5)	84,876 (87.3)	12,312 (88.7)	<0.01^c^
Alcohol consumption	27,475 (24.7)	23,468 (24.1)	4007 (28.9)	<0.01^c^

Values are presented as numbers (percentages) unless otherwise indicated.

Abbreviations: MetS, metabolic syndrome.

a age is presented as the median (interquartile range)

b the Mann-Whitney *U* test

c Chi-square test

**Fig. 2:**
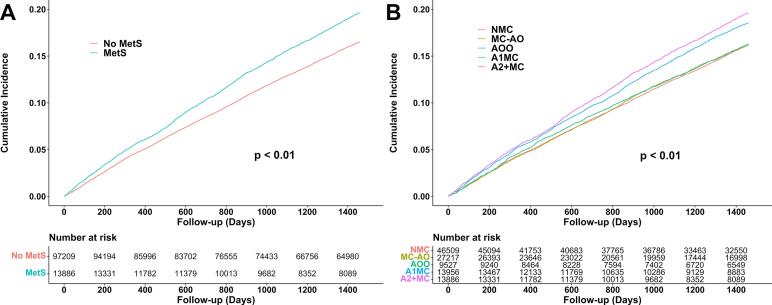
Kaplan–Meier estimates of cumulative incidence of low back pain among participants who underwent the Specific Health Checkup in fiscal year 2018, based on nationwide insurance claims and health checkup data for prefectural government employees in Japan. Panel A: Kaplan–Meier estimates comparing participants with and without metabolic syndrome (log-rank test: *p* < 0.01). Panel B: Kaplan–Meier estimates across groups categorized by different combinations of metabolic syndrome components (log-rank test: p < 0.01). Abbreviations: MetS, metabolic syndrome; NMC, no metabolic syndrome components; MC-AO, metabolic syndrome components without abdominal obesity; AOO, abdominal obesity only; A1MC, abdominal obesity with one additional metabolic syndrome component; A2 + MC, abdominal obesity with two or more metabolic syndrome components.

Fig. 2 illustrates the survival curve. The mean follow-up period was 3.3 years, with a total observation time of 362,911.6 person-years. During the four-year observation period, 16,894 participants developed LBP, representing 15.2 % of the cohort and yielding an overall incidence rate of 46.6 per 1000 person-years. Censoring due to loss of health insurance eligibility occurred in 21,524 participants (19.4 %), and censoring due to death occurred in 139 participants (0.1 %). The incidence rate was higher in the MetS group (55.2 per 1000 person-years) than in the non-MetS group (45.4 per 1000 person-years). Among the five groups with different MetS component combinations, incidence rates (per 1000 person-years) were as follows: 44.3 in those with no MetS components, 44.3 in those with MetS components excluding abdominal obesity, 44.9 in those with abdominal obesity only, 51.7 in those with abdominal obesity with one additional MetS component, and 55.2 in those with abdominal obesity with two or more MetS components.

Table 2 presents the Cox proportional hazards analysis results examining the association between MetS and LBP. The risk of incident LBP was significantly higher in the MetS group than in the non-MetS group (HR = 1.24; 95 % CI, 1.19–1.30). When stratified by sex, the HR was 1.23 (95 % CI, 1.17–1.29) for males and 1.34 (95 % CI, 1.17–1.54) for females. The MetS × sex interaction was not significant (*p* = 0.29). For individual MetS components, hypertension (HR = 1.08; 95 % CI, 1.05–1.12), hyperlipidemia (HR = 1.19; 95 % CI, 1.14–1.23), and hyperglycemia (HR = 1.09; 95 % CI, 1.04–1.15) were modestly associated with LBP. These component-specific estimates are presented in Supplementary Table 3.

**Table 2:** Cox proportional hazards analysis examining the association between metabolic syndrome and low back pain among prefectural government employees in Japan who underwent the Specific Health Checkup in fiscal year 2018.

		n	%	Unadjusted	Adjusted a
				HR (95 % CI)	HR (95 % CI)
Overall	No MetS	97,209	14.9	1.00	1.00
	MetS	13,886	17.1	1.21 (1.16–1.27)	1.24 (1.19–1.30)
Male	No MetS	65,711	14.3	1.00	1.00
	MetS	12,789	16.8	1.25 (1.19–1.31)	1.23 (1.17–1.29)
Female	No MetS	31,498	16.4	1.00	1.00
	MetS	1097	20.3	1.35 (1.18–1.54)	1.34 (1.17–1.54)

Abbreviations: HR, hazard ratio; CI, confidence interval; MetS, metabolic syndrome.

a Adjusted for age, sex, smoking, physical inactivity, and alcohol consumption.

Table 3 presents the analysis results for MetS component combinations. Groups with abdominal obesity exhibited an elevated risk of incident LBP, whereas those with MetS components excluding abdominal obesity showed no significant association (HR = 1.03; 95 % CI, 0.99–1.08). The HR was 1.07 (95 % CI, 1.01–1.14) for those with abdominal obesity only, 1.24 (95 % CI, 1.18–1.30) for those with abdominal obesity with one additional MetS component, and 1.32 (95 % CI, 1.25–1.38) for those with abdominal obesity with two or more MetS components, suggesting a progressive risk increase with additional MetS components.

**Table 3:** Cox proportional hazards analysis examining the association between metabolic syndrome component combinations and low back pain among prefectural government employees in Japan who underwent the Specific Health Checkup in fiscal year 2018.

		N	%	Unadjusted	Adjusted a
				HR (95 % CI)	HR (95 % CI)
Overall	NMC	46,509	14.9	1.00	1.00
	MC-AO	27,217	14.2	1.00 (0.96–1.04)	1.03 (0.99–1.08)
	AOO	9527	14.9	1.01 (0.96–1.07)	1.07 (1.01–1.14)
	A1MC	13,956	16.6	1.17 (1.11–1.22)	1.24 (1.18–1.30)
	A2 + MC	13,886	17.1	1.24 (1.19–1.30)	1.32 (1.25–1.38)
Male	NMC	24,451	13.8	1.00	1.00
	MC-AO	20,696	13.6	1.03 (0.98–1.09)	1.02 (0.97–1.07)
	AOO	8093	14.6	1.07 (1.00–1.15)	1.07 (1.00–1.14)
	A1MC	12,471	16.2	1.23 (1.17–1.30)	1.22 (1.16–1.29)
	A2 + MC	12,789	16.8	1.32 (1.25–1.40)	1.30 (1.23–1.37)
Female	NMC	22,058	16.1	1.00	1.00
	MC-AO	6521	16.2	1.06 (0.99–1.14)	1.06 (0.99–1.14)
	AOO	1434	17.0	1.07 (0.94–1.22)	1.06 (0.93–1.21)
	A1MC	1485	19.8	1.30 (1.15–1.46)	1.29 (1.15–1.46)
	A2 + MC	1097	20.3	1.39 (1.21–1.59)	1.39 (1.21–1.59)

Abbreviations: HR, hazard ratio; CI, confidence interval; NMC, no metabolic syndrome components; MC-AO, metabolic syndrome components without abdominal obesity; AOO, abdominal obesity only; A1MC, abdominal obesity with one additional metabolic syndrome component; A2 + MC, abdominal obesity with two or more metabolic syndrome components.

a Adjusted for age, sex, smoking, physical inactivity, and alcohol consumption.

When stratified by sex, no significant association was found in males with MetS components excluding abdominal obesity (HR = 1.02; 95 % CI, 0.97–1.07), whereas risk consistently increased in those with abdominal obesity: abdominal obesity only (HR = 1.07; 95 % CI, 1.00–1.14); abdominal obesity with one additional MetS component (HR = 1.22; 95 % CI, 1.15–1.29); and abdominal obesity with two or more MetS components (HR = 1.30; 95 % CI, 1.23–1.37). A similar pattern was observed in females: Neither MetS components excluding abdominal obesity (HR = 1.06; 95 % CI, 0.99–1.14) nor abdominal obesity only (HR = 1.06; 95 % CI, 0.93–1.21) was significant; however, risk increased with abdominal obesity with one (HR = 1.29; 95 % CI, 1.15–1.46) and two or more MetS components (HR = 1.39, 95 % CI, 1.21–1.59).

Supplementary Tables 4 and 5 present the sensitivity analysis results, after applying a six-month lag after baseline. The findings from the other sensitivity analyses are summarized in the text. With a six-month lag period after the baseline period, the HR for LBP in participants with MetS was 1.23 (95 % CI, 1.17–1.29). This findings remained consistent with that obtained without a lag period. When sciatica was excluded from the LBP definition, the HR for MetS was 1.23 (95 % CI, 1.18–1.29); when sex-specific HDL-C cut-offs were applied, the HR was 1.24 (95 % CI, 1.19–1.30). Both estimates were virtually unchanged from the primary analysis. The MetS × age interaction was not significant (*p* = 0.35).

## 4 Discussion

In this study, a longitudinal analysis was conducted using large-scale insurance claims and health checkup data from middle-aged Japanese workers and found that MetS was associated with an increased risk of incident LBP. Moreover, among individuals with abdominal obesity, the risk of incident LBP was higher when abdominal obesity co-occurred with other MetS components than when it was present alone.

This study's findings consistent with several cross-sectional research. For example, a study from the United Kingdom (Perera et al., 2022) reported that obese individuals with MetS had a 42 % higher risk of developing LBP than those without MetS. Similarly, a Japanese study (Yoshimoto et al., 2019) found that the accumulation of one or more MetS components alongside abdominal obesity was associated with LBP in females. By contrast, our results did not correlate with the findings of a recent longitudinal study from China (Huang et al., 2024). This inconsistency across studies may reflect differences in the diagnostic criteria for MetS and, importantly, in the definition of LBP (self-reported questionnaire versus claims-based diagnoses). In the Chinese cohort, healthier behaviors—such as lower prevalence of smoking and alcohol consumption in the MetS group—may have also contributed to the discrepant findings.

Several mechanisms have been proposed linking MetS to LBP. First, obesity—one of the MetS components—is a well-established risk factor for LBP (Elgaeva et al., 2020; Lean et al., 1998; Shiri et al., 2010b). Obesity may contribute to mechanical stress on the musculoskeletal system (Rodriguez-Martinez et al., 2016; Singh et al., 2015), physical inactivity (Shiri et al., 2013; Smuck et al., 2014), and inflammation due to excessive fat accumulation (Briggs et al., 2013; Shoelson et al., 2007). Furthermore, dyslipidemia (Leino-Arjas et al., 2006; Yoshimoto et al., 2018) and diabetes (Real et al., 2019) have both been linked to increased LBP risk. Potential mechanisms include atherosclerotic changes that reduce blood flow to lumbar structures, such as intervertebral discs and muscles (Kauppila et al., 1997; Kauppila, 2009), and the promotion of catabolism and inflammation by advanced glycation end products (Allegri et al., 2016). These findings suggest that LBP risk increases when abdominal obesity is combined with metabolic factors.

In this study, no significant interaction between MetS and sex was observed with respect to LBP risk. Unlike our findings, three previous cross-sectional studies from Japan and the United Kingdom (Ono et al., 2012; Perera et al., 2022; Yoshimoto et al., 2019) reported a significant association only in females. One plausible explanation for such sex differences is the decline in estrogen levels, which has been linked with both MetS (Carr, 2003) and reduced intervertebral disc height (Baron et al., 2005), potentially contributing to LBP (Raastad et al., 2015). However, our much larger longitudinal cohort may simply have had greater power to detect the association in males as well. Methodological differences including study design and the methods used to identify and define LBP could also account for these discrepant findings across studies.

This study has several strengths. First, the large sample size minimized potential biases and reduced random error. Moreover, the high health checkup participation rate (85.5 % in fiscal year 2018) (Ministry of Health, Labour and Welfare, 2025b) and the availability of insurance claims data for all participants reduced selection bias. The use of longitudinal insurance claims data to identify LBP also enhanced the validity of LBP risk estimates associated with MetS. Furthermore, as the study participants were middle-aged employees of prefectural governments across Japan (excluding Tokyo), the findings are generalizable to middle-aged Japanese workers.

However, this study has several limitations. First, because LBP was classified using ICD-10 codes from diagnostic information in insurance claims data, misclassification bias is possible. If LBP from causes unrelated to MetS, such as trauma, was included, the risk may have been overestimated. However, diagnosis-based code classification ensures greater accuracy and objectivity than self-reported questionnaires. Second, several factors related to MetS and LBP—including occupational conditions, physical workload, and mental health—were not captured in our dataset. In particular, depressive symptoms, whose co-occurrence with MetS has been linked to an increased risk of LBP (Huang et al., 2024), were not assessed and, therefore, could not be adjusted for. Third, our findings should be extrapolated to the general Japanese population with caution, because our cohort excluded individuals aged 65 years or older and those who had lost health insurance eligibility. Finally, lifestyle data were self-reported, potentially reducing accuracy and objectivity. Consequently, confounding effects may not have been fully adjusted for. Because self-reported behaviors are often under-reported, such nondifferential misclassification could bias the HR estimates toward the null.

## 5 Conclusions

In this large-scale longitudinal study using RWD, MetS was associated with an increased risk of incident LBP among middle-aged Japanese workers. Moreover, individuals with abdominal obesity with additional MetS components faced a higher risk than those with abdominal obesity alone. These findings provide longitudinal evidence supporting the association between MetS and LBP, suggesting that MetS may serve as a predictor of LBP and thus highlighting the potential value of MetS management in LBP prevention.

## CRediT authorship contribution statement

**Shinsuke Okawa:** Writing – review & editing, Writing – original draft, Visualization, Software, Resources, Methodology, Formal analysis, Data curation, Conceptualization. **Takuya Yamada:** Writing – review & editing, Writing – original draft, Resources, Project administration, Methodology, Investigation, Formal analysis, Data curation, Conceptualization. **Mari Irie:** Writing – review & editing, Resources, Methodology, Data curation. **Kumi Sugimoto:** Writing – review & editing, Resources, Project administration, Investigation, Data curation. **Yoshiharu Fukuda:** Writing – review & editing, Supervision, Project administration, Conceptualization.

## Declaration of generative AI and AI-assisted technologies in the writing process

Statement: During the preparation of this work, the authors used ChatGPT to enhance readability and language. After using this service, the authors reviewed and edited the content as needed and take full responsibility for the content of the publication.

## Funding

This research did not receive any specific grant from funding agencies in the public, commercial, or not-for-profit sectors.

## Declaration of competing interest

The authors declare that they have no known competing financial interests or personal relationships that could have appeared to influence the work reported in this paper.

## Data Availability

The data used in this manuscript cannot be shared due to privacy and ethical restrictions.

## Appendix A Supplementary data

Supplementary material Image 1
