# Supplementary material for: Association between metabolic syndrome and risk of incident low back pain: A retrospective cohort study using real-world data from Japan
Source: Prev Med Rep. 2025 Jul 4;56:103162. doi: 10.1016/j.pmedr.2025.103162 (PMC12274325; doi:10.1016/j.pmedr.2025.103162)
Supplement: Supplementary file 1 — Supplementary material [file mmc1.docx]

Supplementary Table 1. Definitions of the five groups based on abdominal obesity and the number of additional metabolic syndrome components

| Category | Abbreviation | Definition |
| --- | --- | --- |
| No metabolic syndrome components | NMC | Participants with none of the metabolic syndrome components  —abdominal obesity, hypertension, hyperlipidemia, or hyperglycemia |
| Metabolic syndrome components  excluding abdominal obesity | MC-AO | Participants with at least one metabolic syndrome component other than  abdominal obesity (hypertension, hyperlipidemia, or hyperglycemia) |
| Abdominal obesity only | AOO | Participants with abdominal obesity but with none of the other metabolic syndrome components |
| Abdominal obesity with one additional  metabolic syndrome component | A1MC | Participants with abdominal obesity and only one of the following:  hypertension, hyperlipidemia, or hyperglycemia |
| Abdominal obesity with two or more  metabolic syndrome components | A2+MC | Participants with abdominal obesity and at least two of the following:  hypertension, hyperlipidemia, or hyperglycemia |

Supplementary Table 2. Baseline characteristics of participants based on combinations of metabolic syndrome component among prefectural government employees in Japan who underwent the Specific Health Checkup in fiscal year 2018

| Variable | Overall (n =111,095) | NMC (n = 46,509) | MC-AO  (n = 27,217) | AOO (n = 9,527) | A1MC (n = 13,956) | A2+MC (n = 13,886) | p-value |
| --- | --- | --- | --- | --- | --- | --- | --- |
| Demographics |  |  |  |  |  |  |  |
| Age – years ^a^ | 50.0 (46.0–55.0) | 48.0 (44.0–53.0) | 52.0 (47.0–56.0) | 49.0 (45.0–54.0) | 51.0 (47.0–55.0) | 53.0 (49.0–57.0) | <0.01^b^ |
| Sex |  |  |  |  |  |  | <0.01^c^ |
| Male | 78,500 (70.7) | 24,451 (52.6) | 20,696 (76.0) | 8,093 (84.9) | 12,471 (89.4) | 12,789 (92.1) |  |
| Female | 32,595 (29.3) | 22,058 (47.4) | 6,521 (24.0) | 1,434 (15.1) | 1,485 (10.6) | 1,097 (7.9) |  |
| Health Behaviors |  |  |  |  |  |  |  |
| Smoking | 16,693 (15.0) | 4,681 (10.1) | 4,508 (16.6) | 1,563 (16.4) | 2,763 (19.8) | 3,178 (22.9) | <0.01^c^ |
| Physical inactivity | 97,188 (87.5) | 40,937 (88.0) | 23,278 (85.5) | 8,404 (88.2) | 12,257 (87.8) | 12,312 (88.7) | <0.01^c^ |
| Alcohol consumption | 27,475 (24.7) | 8,812 (18.9) | 8,564 (31.5) | 2,234 (23.4) | 3,858 (27.6) | 4,007 (28.9) | <0.01^c^ |

Values are presented as numbers (percentages), unless otherwise indicated.

^a^ age is presented as median (interquartile range); ^b^  Kruskal-Wallis test; ^c^ Chi-square test

Abbreviations: NMC, no metabolic syndrome components; MC-AO, metabolic syndrome components without abdominal obesity; AOO, abdominal obesity only; A1MC, abdominal obesity with one additional metabolic syndrome component; A2+MC, abdominal obesity with two or more metabolic syndrome components

Supplementary Table 3. Cox proportional hazards analysis examining the association between individual metabolic syndrome components and low back pain among prefectural government employees in Japan who underwent the Specific Health Checkup in fiscal year 2018

|  |  | n | % | Unadjusted | Adjusted ^a^ |
| --- | --- | --- | --- | --- | --- |
|  |  |  |  | HR (95% CI) | HR (95% CI) |
| Overall | hypertension | 38,992 | 15.4 | 1.07 (1.04–1.10) | 1.08 (1.05–1.12) |
|  | hyperlipidemia | 28,265 | 16.4 | 1.16 (1.12–1.20) | 1.19 (1.14–1.23) |
|  | hyperglycemia | 13,511 | 15.4 | 1.09 (1.04–1.14) | 1.09 (1.04–1.15) |
| Male | hypertension | 32,460 | 15.0 | 1.09 (1.05–1.13) | 1.08 (1.04–1.12) |
|  | hyperlipidemia | 24,698 | 16.1 | 1.19 (1.14–1.24) | 1.18 (1.13–1.23) |
|  | hyperglycemia | 11,817 | 15.2 | 1.12 (1.06–1.17) | 1.09 (1.04–1.15) |
| Female | hypertension | 6,532 | 17.2 | 1.11 (1.04–1.19) | 1.11 (1.04–1.19) |
|  | hyperlipidemia | 3,567 | 18.6 | 1.23 (1.13–1.35) | 1.22 (1.13–1.33) |
|  | hyperglycemia | 1,694 | 16.8 | 1.09 (0.97–1.23) | 1.08 (0.96–1.22) |

Abbreviations: HR, hazard ratio; CI, confidence interval

^a^ Adjusted for age, sex, smoking, physical inactivity, and alcohol consumption.

Supplementary Table 4. Cox proportional hazards analysis examining the association between metabolic syndrome and low back pain with a six-month lag period after the baseline period among prefectural government employees in Japan who underwent the Specific Health Checkup in fiscal year 2018

|  |  | n | % | Unadjusted | Adjusted ^a^ |
| --- | --- | --- | --- | --- | --- |
|  |  |  |  | HR (95% CI) | HR (95% CI) |
| Overall | No MetS | 94,395 | 12.9 | 1.00 | 1.00 |
|  | MetS | 13,376 | 14.6 | 1.20 (1.14–1.26) | 1.23 (1.17–1.29) |
| Male | No MetS | 63,889 | 12.3 | 1.00 | 1.00 |
|  | MetS | 12,321 | 14.3 | 1.23 (1.17–1.30) | 1.21 (1.15–1.28) |
| Female | No MetS | 30,506 | 14.3 | 1.00 | 1.00 |
|  | MetS | 1055 | 17.4 | 1.34 (1.15–1.55) | 1.32 (1.14–1.53) |

Abbreviations: HR, hazard ratio; CI, confidence interval, MetS; metabolic syndrome

^a^ Adjusted for age, sex, smoking, physical inactivity, and alcohol consumption.

Supplementary Table 5. Cox proportional hazards analysis examining the association between metabolic syndrome component combinations and low back pain with a six-month lag period after the baseline period among prefectural government employees in Japan who underwent the Specific Health Checkup in fiscal year 2018

|  |  | n | % | Unadjusted | Adjusted ^a^ |
| --- | --- | --- | --- | --- | --- |
|  |  |  |  | HR (95% CI) | HR (95% CI) |
| Overall | NMC | 45,192 | 12.9 | 1.00 | 1.00 |
|  | MC-AO | 26,446 | 12.2 | 1.00 (0.96–1.04) | 1.03 (0.98–1.08) |
|  | AOO | 9,255 | 12.9 | 1.01 (0.95–1.08) | 1.07 (1.00–1.14) |
|  | A1MC | 13,502 | 14.2 | 1.15 (1.09–1.21) | 1.22 (1.16–1.29) |
|  | A2+MC | 13,376 | 14.5 | 1.23 (1.17–1.29) | 1.30 (1.23–1.37) |
| Male | NMC | 23,824 | 12.0 | 1.00 | 1.00 |
|  | MC-AO | 20,123 | 11.6 | 1.02 (0.97–1.08) | 1.01 (0.95–1.06) |
|  | AOO | 7,865 | 12.5 | 1.06 (0.99–1.14) | 1.06 (0.98–1.14) |
|  | A1MC | 12,077 | 13.9 | 1.22 (1.14–1.29) | 1.20 (1.13–1.27) |
|  | A2+MC | 12,321 | 14.3 | 1.30 (1.23–1.38) | 1.27 (1.20–1.35) |
| Female | NMC | 21,368 | 14.0 | 1.00 | 1.00 |
|  | MC-AO | 6,323 | 14.2 | 1.09 (1.01–1.17) | 1.08 (1.00–1.16) |
|  | AOO | 1,390 | 15.0 | 1.09 (0.94–1.25) | 1.08 (0.94–1.24) |
|  | A1MC | 1,425 | 17.1 | 1.29 (1.13–1.47) | 1.28 (1.12–1.46) |
|  | A2+MC | 1,055 | 17.4 | 1.38 (1.19–1.61) | 1.37 (1.18–1.59) |

Abbreviations: HR, hazard ratio; CI, confidence interval; NMC, no metabolic syndrome components; MC-AO, metabolic syndrome components without abdominal obesity; AOO, abdominal obesity only; A1MC, abdominal obesity with one additional metabolic syndrome component; A2+MC, abdominal obesity with two or more metabolic syndrome components

^a^ Adjusted for age, sex, smoking, physical inactivity, and alcohol consumption.
